# Serum neopterin is elevated in patients infected with *Shigella*

**DOI:** 10.1186/1757-4749-2-9

**Published:** 2010-08-22

**Authors:** Kirnpal-Kaur Banga Singh, WA Wan-Nurfahizul-Izzati, Asma Ismail

**Affiliations:** 1 Institute for Research in Molecular Medicine (INFORMM), Universiti Sains Malaysia, Health Campus, 16150 Kubang Kerian, Kelantan, Malaysia; 2 Department of Medical Microbiology & Parasitology, School of Medical Sciences, Health Campus, Universiti Sains Malaysia, 16150 Kubang Kerian, Kelantan, Malaysia

## Abstract

**Background:**

Neopterin is produced by human macrophages/monocytes when stimulated with interferon-gamma. Production of neopterin is found in serum, cerebrospinal fluid (CSF) and urine of patients with infections by viruses, intracellular bacteria and parasites, autoimmune diseases, malignant tumors and patients in allograft rejection episodes.

**Methods:**

In this study, the level of neopterin was determined in serum samples obtained from patients infected with *Shigella *(all four species) and healthy individuals. The study population comprised of 14 patients infected with *Shigella *and 14 normal controls. Serum neopterin was measured using an enzyme-linked immunosorbent assay (ELISA).

**Results:**

The mean of serum neopterin concentration was 36.32 ± 9.71 nmol/L among patients infected with *Shigella *and 2.88 ± 0.77 nmol/L in the control group. The mean serum neopterin levels were significantly higher in the test group as compared to the normal group (p = 0.002).

**Conclusion:**

This study revealed that neopterin was elevated in patients infected with *Shigella*.

## Introduction

Neopterin is produced by human monocytes/macrophages upon stimulation with interferon gamma produced by activated T-lymphocytes and Natural Killer (NK) cells [1,2]. It is a low molecular weight pteridine compound and is thought to represent a marker of immune activation and the proliferation of macrophages [3]. Change in concentration of neopterin serves as a valuable indicator of clinical progression of the disease to severe, acute form [4].

Levels of neopterin are increased in infections caused by viruses, intracellular bacteria and parasites, autoimmune and other inflammatory diseases, malignant tumors and allograft transplantation [1,2]. Neopterin levels in serum or urine usually correlate with the clinical course. The level is high early in the infection and it decreases as soon as antibodies against the pathogen become detectable [5,6]. Serial measurements of the level of neopterin in a particular patient may be useful in monitoring the course of a condition [4]. Levels of neopterin in body fluids can be determined by high performance liquid chromatography (HPLC), radioimmunoassay (RIA) or ELISA.

*Shigella *spp. is the major cause of bacillary dysentery worldwide. It is a Gram negative, non-motile, non-spore-forming rod-shaped bacterium that is highly infectious. *Shigella *belongs to the family enterobacteriaceae and it is transmitted *via *contaminated food and through fecal-oral route. Annually, worldwide episodes of *Shigella *have been estimated to be around 164.7 million, among which 163.2 million episodes occur in developing countries and are associated with 1.1 million deaths, mostly among children under 5 years old [7]. Shigellosis is an invasive disease of the human intestines and is characterized by passage of frequent small-volume of loose stool, consisting largely of blood and mucus. Symptoms of shigellosis include presentation with fever, abdominal pain and cramps. Early diagnosis of *Shigella *is important as it has very low (10 to 100 organisms) infective dose [8].

As *Shigella *spp. are intracellular organisms, the aim of this study was to determine whether neopterin could be used as a marker of macrophage activation in patients infected with this organism. We examine the concentration of neopterin in healthy individuals and patients infected with all four *Shigella *spp. The levels of neopterin in serum were determined using ELISA.

## Materials and methods

### Sera

Sera were collected after informed consents from patients admitted to the Hospital Universiti Sains Malaysia, Kubang Kerian, Kelantan, Malaysia. The sera (n = 28) were from patients with shigellosis confirmed by stool culture (n = 14) and from healthy individuals who did not have diarrhea since past six months (n = 14). All the samples were collected from patients and individuals who did not have any concurrent chronic or autoimmune diseases. All the collected sera were stored in -20°C until measurements were carried out.

### ELISA procedure

For quantitative determinations of neopterin in serum, competitive enzyme immunosorbent assay (ELISA) was used (neopterin ELISA, IBL, Hamburg). The test was performed according to the protocol from the manufacturer. The intensity of the color developed and the absorbance was found to be inversely proportional to the amount of neopterin present in the samples. Thus, the higher the neopterin concentration in the sample, the lower was the A450 value(s) we obtained. Standards of 0.00, 1.35, 4.00, 12.00, 37.00 and 111.00 nmol/L were used to prepare a standard curve. The levels of neopterin concentration in the samples were determined based on the standard curve.

### Statistical evaluation

The statistical significance of the data was determined by the independent Student's t-test using SPSS version 15 (SPSS Inc., USA). The differences between the groups were taken as statistically significant when p < 0.05.

## Results and Discussion

**Figure 1:**
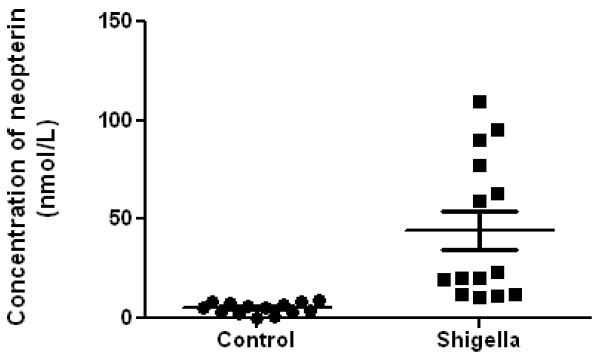
**Serum concentration of neopterin in healthy individuals (n = 14) and patients infected with *Shigella *(n = 14) using ELISA test (p = 0.02)**.

Our observations as depicted in Figure 1 reveal concentrations of neopterin in patient sera infected with *Shigella *and their comparison with healthy individuals. In infected patients, the concentration of neopterin obtained in this study was >10 nmol/L whereas in healthy individuals, the concentration of neopterin was < 10 nmol/L. The mean concentration of serum neopterin levels were found to be 36.32 ± 9.71 nmol/L in patients infected with *Shigella *as compared to 2.88 ± 0.77 nmol/L in the control group. Serum neopterin levels were significantly elevated in patients with shigellosis compared to the control group (p = 0.002)

Previous studies indicated that neopterin levels can be detected in morbidities such as lung infections, HIV infection, hepatitis B infection, sarcoidosis as well as in others chronic diseases [1,5,9-11]. To date, there is no published report describing elevated levels of neopterin in shigellosis or any other acute diarrheal diseases. In this study, serum neopterin levels were found to be significantly higher in patients infected with *Shigella *compared to healthy individuals. The results of this study are comparable to the previous studies in which the levels of neopterin were elevated in patients infected with intracellular pathogens (> 10 nmol/L) compared to healthy individuals (< 10 nmol/L) [1]. As *Shigella *is an intracellular pathogen, the cellular immune system would be activated and immunological processes could have been triggered by endotoxins produced by Gram negative bacteria. Consequently, the activation of T-lymphocytes and formation of INF-gamma could increase the concentration of neopterin in body fluids.

Previous studies have shown that highest concentrations of neopterin in body fluids were detected in cases of septic complications and due to LPS from Gram negative bacteria [12]. The important aspect of our study was the discriminating ability of neopterin detection between patients infected with *Shigella *and healthy individuals. Despite the fact that neopterin was detected in the control serum, their levels were still lower compared to the lowest level of neopterin in shigellosis patient's serum. We believe that this is the first report describing significant increase of neopterin in patients infected with *Shigella *compared to healthy individuals. The obvious limitation of this study is due to the relatively small size of the patient population investigated. Nonetheless, our study should be seen as the first observation that suggests the use of serum neopterin levels as a possible, clinical level, surrogate marker of shigellosis.

## Competing interests

The authors declare that they have no competing interests.

## Authors' contributions

WWA carried out the experiments and drafted the manuscript. AI participated in the design of the study and edited the manuscript. KBS participated in the acquisition of funding, design of the study, coordination and monitoring of research, and edited the manuscript. All authors have read and approved the final manuscript.
